# Efficacy of biological agents and fillers seed coating in improving drought stress in anise

**DOI:** 10.3389/fpls.2022.955512

**Published:** 2022-07-22

**Authors:** Atefeh Hoseini, Amin Salehi, R. Z. Sayyed, Hamidreza Balouchi, Ali Moradi, Ramin Piri, Bahman Fazeli-Nasab, Peter Poczai, Mohammad Javed Ansari, Sami Al Obaid, Rahul Datta

**Affiliations:** ^1^Department of Agronomy and Plant Breeding, Yasouj University, Yasouj, Iran; ^2^Department of Microbiology, PSGVP Mandal’s S I Patil Arts, G B Patel Science, and STKV Sangh Commerce College, Shahada, India; ^3^Department of Agronomy and Plant Breeding, Faculty of Agriculture, University of Tehran, Tehran, Iran; ^4^Department of Agronomy and Plant Breeding, Agriculture Institute, Research Institute of Zabol, Zabol, Iran; ^5^Finnish Museum of Natural History, University of Helsinki, Helsinki, Finland; ^6^Department of Botany, Hindu College, (Mahatma Jyotiba Phule Rohilkhand University, Bareilly), Moradabad, India; ^7^Department of Botany and Microbiology, College of Science, King Saud University, Riyadh, Saudi Arabia; ^8^Department of Geology and Pedology, Mendel University in Brno, Brno, Czechia

**Keywords:** anise, drought stress, *Pseudomonas fluorescent*, seed coating, *Trichoderma harzianum*

## Abstract

Many plants, including anise, have tiny, non-uniform seeds with low and light nutrient reserves. The seeds also show a weak establishment, especially under stressful conditions where their accurate planting in the soil and optimal yield are tough. This study sought to improve anise seeds’ physical and physiological characteristics under drought stress. To this end, two factorial experiments under laboratory and greenhouse conditions were performed in a completely randomized design with 4 and 3 replications, respectively. Five levels of seed inoculation (inoculation with T36 and T43 of *Trichoderma harzianum*, and CHA0 and B52 of *Pseudomonas fluorescent*, and non-inoculation which means that control seeds were not treated with microbial inoculant), three levels of coating (K10P20, K10P10V5, and non-coating), and three levels of drought stress (0, –3, and –6 bars) were considered as the factorial experiment [vermiculite (V), kaolin (K), and perlite (P) numbers refer to the amount of material used in grams]. The laboratory experiment revealed that the combined treatments of bio-agents with coating increased the physical and germination characteristics of anise seeds compared to the control treatment. The greenhouse experiment showed that drought stress reduced the initial growth indices. Still, the combination treatments of biological agents and coating (fillers) could alleviate the destructive effects of drought stress to some extent and improve these indices. The best treatment was provided by T36 and K10P20 in both experiments, which significantly increased morphological indices.

## Highlights

- Biological agents and seed coating increased the germination indices of anise seeds.
- Biological agents and seed coating ameliorate the destructive effects of drought stress.
- In both experiments, the best treatment was the combination of T36 and K10P20.

## Introduction

High-quality seeds in agriculture cause uniform and rapid emergence of seedlings, higher plant establishment, greater density, and, thereby increased yields (Finch-Savage and Bassel, 2016; Caverzan et al., 2018; Bright et al., 2022). Seed germination is one of the seed quality parameters that plays a decisive role in proper seedling establishment and subsequent success in later stages of plant growth. Anise (*Pimpinella anisum* L.) is an annual medicinal plant with numerous medical uses and antibacterial, antispasmodic, antiviral, fungicidal, insecticidal, and anti-pneumonia qualities. This plant has low vigor, vitality, and storage materials. Therefore, germination and planting of its seedlings are weak, and it is susceptible to environmental stresses in these stages (Bettaieb Rebey et al., 2018; Sun et al., 2019).

Environmental factors, especially drought stress, can affect seed germination and seedling growth in such a way that activation of enzymes can be prevented in the germination stage, and plant life cycle can be disrupted in this sensitive stage (Rezayian et al., 2018; Khan et al., 2019; Kour et al., 2019; Mahpara et al., 2022). Different studies have shown that drought stress impacts the seed germination of various plants (Naseem et al., 2018; Khan and Bano, 2019; Zhang et al., 2019). For instance, it was reported that anise and fennel germination and seedling indices were reduced under drought stress (Farhoudi and Khordahampour, 2017). In another study, Aćimović et al. (2014); Shatpathy et al. (2018), Hojjat (2020), and Ilyas et al. (2020), it was claimed that drought stress reduced total germination energy in anise seeds.

Seed coating with biological agents is one of the enhancement methods that can improve tolerance to stresses and enhance seed germination, seedling indices, and growth efficiency in different plants (Ma et al., 2019; Rocha et al., 2019). Furthermore, seed coating with aims such as creating a favorable environment for seed establishment and better growth using growth stimulants and nutrients, facilitating seed sowing (possibility of mechanized planting), utilizing biological agents, and reducing the use of chemical fertilizers, and applying moisture-absorber materials, is employed to deal with drought stress (Su et al., 2017). Similarly, moisture-absorbing minerals and polymeric adhesives such as gum Arabic, exopolysaccharides (Ilyas et al., 2020), and biofilms (Fazeli-Nasab et al., 2022a; Sheikh et al., 2022) can increase the survival of microorganisms in seed inoculation by moisture uptake (Javed et al., 2022).

Drought stress reduces the germination and seedling indices of crop plants, whereas PGPR could improve these indices under stress conditions (Kasim et al., 2013; Fazeli-Nasab and Sayyed, 2019). *Trichoderma harzianum* was also found to increase germination percentage and seedling indices under laboratory and greenhouse conditions (Guler et al., 2016). In this regard, it was concluded that inoculation of corn seeds with *Trichoderma* fungus increased seedlings’ fresh and dry weights under drought stress. In another study (Piri et al., 2020), the inoculation of seeds with *Pseudomonas* spp. and other PGPR improved the seed germination and seedling indices of various crops under salinity stress (Fazeli-Nasab and Sayyed, 2019; Kapadia et al., 2021; Khan et al., 2021). Piri et al. (2019) assessed the effects of seed inoculation and coating on cumin (*Cuminum cyminum*) seedling indices under drought stress. Their results demonstrated that coating the seeds of cumin with moisture-absorbing compounds, *Pseudomonas* bacteria, and *Trichoderma* fungus improved the root and shoot length and subsequently improved seedling performance under drought stress.

PGPR promotes plant growth through various mechanisms (Kalam et al., 2020) like production of phytohormones (Sheikh et al., 2022) and siderophores (Nithyapriya et al., 2021), phosphate solubilization (PS) (Sayyed et al., 2019), and exo-polysaccharide production (Sheikh et al., 2022). Siderophores enhance the iron uptake of plants by formation of a complex with an insoluble form of iron (ferric) in the soil and is converted later to a ferrous form in plants (Patel et al., 2018).

Due to the fact that anise is a plant with low seed vigor and fine seeds that make the establishment of this plant difficult, especially in drought stress, the purpose of this research was to evaluate the effect of bio-priming and coating on the seedling vigor and germination indices of anise under drought stress.

## Materials and methods

### Experimental design

Seeds of anise were collected from the Friedan area of Isfahan province, Iran. Before the beginning of the experiment, seed viability was assessed by tetrazolium test (seed viability = 97%). To obtain the optimal composition of the coating, a pre-experiment with 29 coating treatments and a combination of vermiculite (V), kaolin (K), and perlite (P) was performed in a CRD (completely randomized design) with four replications. Also, numbers refer to the amount of material used in grams (Piri et al., 2019).

Laboratory experiments were conducted to investigate the effects of the combination of coating and microbial inoculants on anise germination and seedling germination indices, as factorials, based on the CRD with four replications. The treatments in this experiment included seed inoculation at five levels (inoculation with T36 and T43 of *T. harzianum* and CHA0 and B52 of *P. fluorescent*, and non-inoculation) and three coating levels (K10P20, K10P10V5, and non-coating) (Piri et al., 2019).

In this regard, a factorial experiment was performed based on the CRD with three replications in the greenhouse to investigate the impact of bio-priming and seed coating on the total growth and initial growth indices of anise seedlings under drought stress. Five levels of seed inoculation (including inoculation with T43 and T36 of *T. harzianum* and CHA0 and B52, of *P. fluorescent*, and non-inoculation), three levels of coating (K10P20, K10P10V5, and Non-coating), and three levels of drought stress (0, –3, and –6 bars) were considered as the factorial experiment.

### Media preparation

#### Fungi

To provide the fungi suspension, the culture medium potato dextrose agar (PDA) (Source: Merck KGaA) was prepared, autoclaved at 121^°^C, and then poured into a Petri dish. After cooling, fungal colony (isolate numbers T36 and T43, *T. harzianum*, accession number: 30,101) samples, respectively, from Golestan province-Nasrabad (35^°^, 50′,07″ N, 54^°^,30′,07″ E) and Mazandaran province-Golidagh (37^°^, 63′,33″ N, 55^°^,94′,70″ E) regions of Iran were cultured in the PDA medium and incubated at 27^°^C for 7 days with the aim of complete sporulation. Finally, for seed inoculation, the suspension concentration with a population of 10^7^ spores per ml of distilled water was adjusted and measured using a hemocytometer under a light microscope (Harman et al., 2008).

#### Bacteria

First, a culture medium, nutrient agar (NA) (Source: Merck KGaA) was prepared and sterilized at 121^°^C, poured into a Petri dish, and cooled. Bacterial isolates (B52 and CHA0 strains of *P. fluorescence*, ATCC 13525) from Shiraz-Fars province and Tehran-Tehran province of Iran) were cultured in the NA in the zigzag shape with a loop test tube and incubated at 28^°^C for 48 h. The absorption of the bacterial suspension was measured at 600 nm and set to 0.5 (10^8^ colonies per ml) (Weller and Cook, 1983).

Before inoculation, the seeds were superficially disinfected with 2% hypochlorite. Next, they were immersed in 20 ml of a bacterial and fungal suspension (for inoculated treatments) at room temperature (20–25^°^C) for 3 h. Following the inoculation, the seeds were immediately poured into a coating machine, and 0.3% Arabic gum was used to attach the coating composition to the seeds.

### Measurement of plant growth parameters

After applying the inoculation treatments, 30 seeds were sown on a two-layer filter paper in 90-mm Petri dishes. The seeds were incubated for 20 days at 20–30^°^C. At the end of the experiment, the germination indices were measured using Equation 1 (Abdul-Baki and Anderson, 1973), Equation 2 (Verma et al., 2005), and Equation 3 (Reddy and Khan, 2001):


(1) $$\mathrm{Emergence}\,\mathrm{Percentages}=\frac{\text{To}\,\mathrm{tal}\,\mathrm{number}\,\mathrm{of}\,\mathrm{germinated}\,\text{seeds}}{\text{To}\,\mathrm{tal}\,\mathrm{number}\,\mathrm{of}\,\text{seeds}}\times 100$$

(2) $$\mathrm{Germination}\,\mathrm{Rater}=\Sigma \,\frac{\mathrm{Ni}}{\text{Ti}}$$

where

Ni = the number of germinated seeds of days and Ti = the number of days,


(3) See⁢dling⁢Vigor=ling⁢Vigor⁢Raterminated⁢See⁢dling⁢length

In the greenhouse experiment (Tm = 25 ± 2^°^C, humidity = 75 ± 5), soil and sand were used at a 1:1 ratio as the culture media. After applying the inoculation and coating treatments, 20 seeds (coated + inoculated) were planted in pots with 1 kg weight at a depth of 1–2 cm and were irrigated daily. Drought stress has been applied with a polyethylene glycol solution where the required polyethylene glycol was calculated using Michel and Kaufmann’s (1973) method and Equation (4):


Ψs=(1.8×10)-2C-(1.8×10)-4

(4) C+2(2.67×10)-4CT+(8.39×10)-7C2

where Ψ_*S*_ is osmotic pressure in bar, C denotes the concentration of PEG-6000 in g/kg H_2_O, and T shows the temperature in centigrade.

The seedlings were grown in the greenhouse at ± 22^°^C for 4 weeks. The percentage and rate of seedling emergence, meantime of emergence, root length, and shoot length of the seedlings were measured with Equation 5 (Egli and Tekrony, 1995), Equation 6 (Bodsworth and Bewley, 1981), and Equation 7 (Ellis and Roberts, 1981):


(5) $$\mathrm{Emergence}\,\mathrm{Percentage}=\frac{\text{ET}}{\text{ES}}\times 100$$

ES: Emergence seedling;

TS: number of seeds sown


(6) Emergence⁢rate=Σ⁢(En/Dn)

Dn: Day *n* of emergence;

En: number of seedlings that emerged on day *n*


(7) Mean⁢seedling⁢emergence⁢time=Σ⁢(Ni×Ti)/N

Ni = number of seedlings that emerged on the day,

Ti = days after the start of the test,

N = total number of seedling emergence

### Statistical analysis

A data analysis (two-way ANOVA) was performed using SAS software (SAS 9.1). Means were compared by HSD test at a probability level of 5%, and graphs were drawn using Excel.

## Results

### Pre-experiment

**FIGURE 1:**
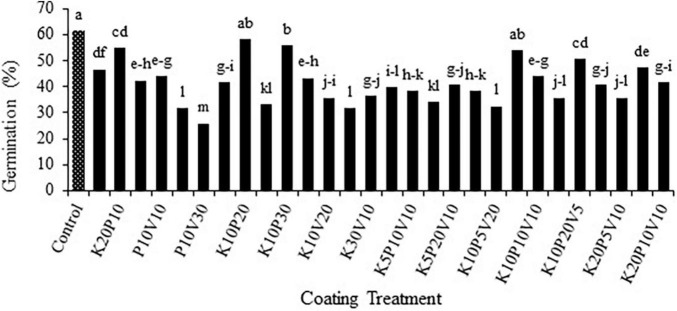
Mean comparison of the effect of seed coating on germination percentage of anise seeds. Means with the same letter are not significantly different according to the HSD test at *P* = 0.05. K, kaolin; V, vermiculite; P, perlite.

The pre-experiment indicated that the most significant germination percentage (61.66%) was obtained from the uncoated treatment. Afterward, coating treatments with the combined ratios of K10P20 and K10 P10V5 (58.33 and 55%, respectively) were selected as the optimal coating treatments. These coating treatments were found to lack any significant difference from the non-coating treatments (Figure 1).

### Laboratory experiment

#### Germination percentage and germination rate

**FIGURE 2:**
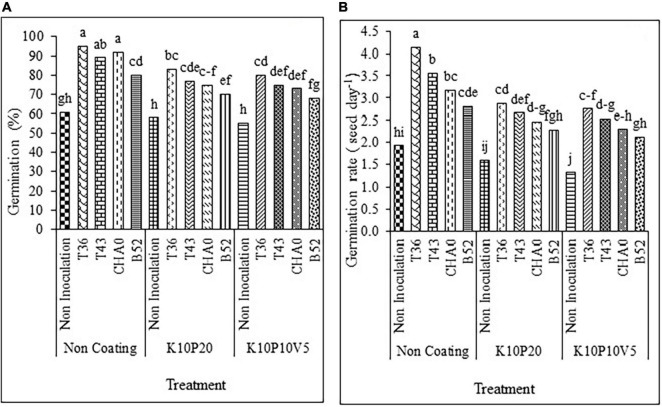
Interaction of **(A)** germination percentage and **(B)** germination rate (seed d^– 1^) of anise affected by inoculation + coating treatments. Means with the same letter are not significantly different according to the HSD test at *P* = 0.05. CHA0, B52 of *Pseudomonas fluorescens*, T36 and T43 of *Trichoderma harzianum* isolates. K, kaolin; V, vermiculite; P, Perlite.

The results revealed that seed coating and inoculation treatments affected the germination percentage of anise seeds (*p* < 0.01). With increase in the coating material (filler coating is a substance used to increase seed size and to carry microorganisms around seeds), seed germination was reduced compared to seeds without coating. A significant difference was also observed between seeds inoculated with fungi and bacteria at all coating levels in such a way that the inoculation of seeds significantly increased the germination percentage compared to the non-inoculated seeds. As it can be seen for all the three coating levels, the highest and lowest germination percentages belonged to the T36 and non-inoculation treatments, respectively (Figure 2A).

The mean comparison demonstrated that the germination rate of the coating treatments was decreased compared to that of the non-coating treatment. However, the different inoculation treatments caused a significant increase in germination rate compared to the control (non-inoculation non-coating) and non-inoculation coating treatments. Similar to germination percentage, the combined treatment of T36 and non-coating, among all the treatments, had the highest germination rate. In contrast, the least germination rate pertained to the combined treatment of non-inoculation and K10P10V5 (Figure 2B).

#### Seedling length

**FIGURE 3:**
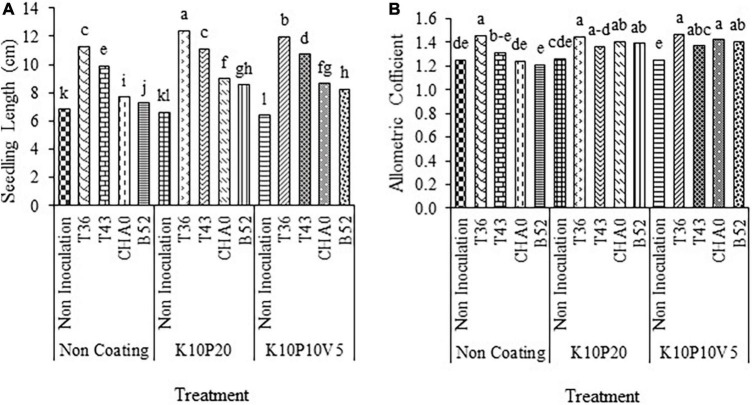
Interaction of **(A)** seedling length (in cm) and **(B)** allometric coefficient of anise affected by inoculation + coating treatment. Means with the same letter are not significantly different according to the HSD test at *P* = 0.05. CHA0, B52 of *Pseudomonas fluorescens*, T36 and T43 of *Trichoderma harzianum* isolates. K, kaolin; V, vermiculite; P, perlite.

The seedling length of anise was significantly affected by the inoculation, coating treatments, and interaction between them (*p* < 0.01). The results indicated that the highest and lowest seedling lengths were observed in T36 and non-inoculated seeds at all coating levels, significantly different from the highest and lowest ones in the other treatments (Figure 3A).

#### Allometric coefficient

The comparison of means regarding the effect of different bio-priming treatments at each coating level showed that the allometric coefficient was significantly increased in bio-priming treatments compared to non-inoculated seeds. Moreover, the T36 treatment at each coating level had the highest value, a statistically significant difference with other treatments. Similar to the other indices, adding a bio-agent to the coating treatments increased the allometric coefficient (Figure 3B).

#### Seedling vigor

**FIGURE 4:**
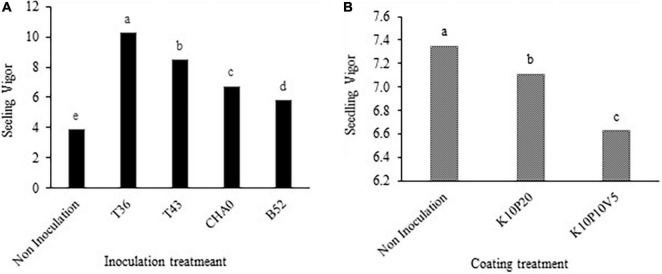
Mean comparison of the effect of **(A)** seed inoculation and **(B)** seed coating on the seedling vigor of anise seeds. Means with the same letter are not significantly different according to the HSD test at *P* = 0.05. CHA0, B52 of *Pseudomonas fluorescens*, T36 and T43 of *Trichoderma harzianum* isolates. K, kaolin; V, vermiculite; P, perlite.

A comparison of means between the different inoculations indicated that the highest seedling vigor (10.27) and the lowest one (3.85) were obtained for the T36 and non-inoculation treatments, respectively (Figure 4A).

In the same way, among the coating treatments, the highest and lowest values of vigor were observed in the non-coating and K10P10V5 treatments, respectively (Figure 4B).

### Greenhouse experiment

#### Seedling emergence percentage and rate

**FIGURE 5:**
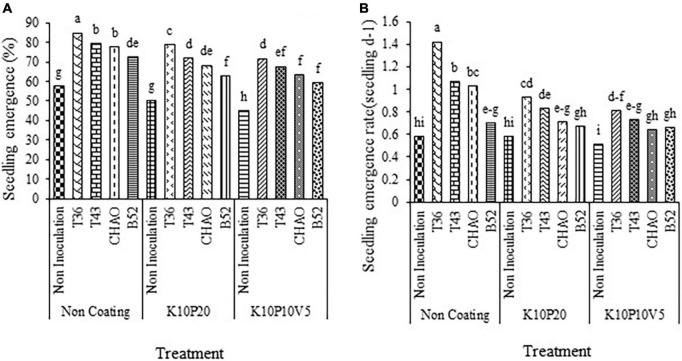
Interaction of **(A)** seedling emergence percentage and **(B)** seedling emergence rate (seedling d^– 1^) of anise affected by inoculation + coating treatments. Means with the same letter are not significantly different according to the HSD test at *P* = 0.05. CHA0, B52 of *Pseudomonas fluorescens*, T36 and T43 of *Trichoderma harzianum* isolates. K, kaolin; V, vermiculite; P, perlite.

The highest percentage of seedling emergence was observed after the T36 treatment (89.44%) at all levels of drought stress, while the lowest (47.22%) was evident after the non-inoculation treatment. The inoculation treatments increased the seedling emergence percentage by 37.2% compared to the non-inoculation treatment (Figure 5A).

The impact of inoculation and the filler coating treatments on seedling emergence rate revealed that the highest value of seedling emergence rate was related to the combination of the T36 and non-coating treatments (1.4 seedling d^–1^) and the lowest (0.5 seedling d^–1^) pertained to the combination of non-inoculation and K10P10V5 (Figure 5B). The means of seedling emergence rate comparison showed that the bio-priming treatments significantly increased this trait compared to the non-inoculated seeds.

**FIGURE 6:**
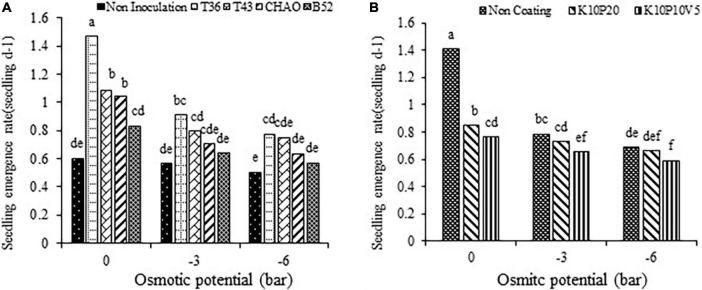
Mean comparison of the effect of **(A)** seed inoculation and **(B)** seed coating on seedling emergence rate (seedling d^– 1^) of anise at different levels of drought stress. Means with the same letter are not significantly different according to the HSD test at *P* = 0.05. CHA0, B52 of *Pseudomonas fluorescens*, T36 and T43 of *Trichoderma harzianum* isolates. K, kaolin; V, vermiculite; P, perlite.

At all levels of drought stress, the highest emergence rate was obtained with the T36 treatment at the 0 bar, while the lowest was obtained with the non-inoculated treatment at the –6 bar (Figure 6A). Moreover, the highest seedling emergence rate was related to the non-coating treatment at the stress of 0 bar (1.40 seedling d^–1^), whereas the lowest seedling emergence (0.76 seedling d^–1^) was related to non-coating treatment at −6 bar. In general, with increase in drought stress, the seedling emergence rate decreased, so the lowest emergence rate was observed at the –6 bar (Figure 6B).

#### Mean of seedling emergence time

**FIGURE 7:**
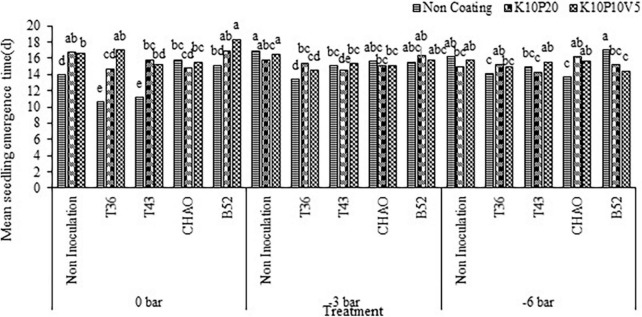
Interaction of mean seedling emergence time (d) of cumin affected by inoculation + coating at different levels of drought stress. Means with the same letter are not significantly different according to the HSD test at *P* = 0.05. CHA0, B52 of *Pseudomonas fluorescens*, T36 and T43 of *Trichoderma harzianum* isolates. K, kaolin; V, vermiculite; P, perlite.

The mean of seedling emergence time was significantly affected by coating, inoculation, and drought stress treatments, and interaction between them (*p* < 0.01). In contrast to the seedling emergence rate, the mean of seedling emergence time increased with drought stress level in such a way that the stress level increased from 0 bar and 13.9 days to –6 bar and 16.94 days in the non-coating non-inoculation (control) treatment. At the 0 bar, the combination of B52 and K10P10V5 treatments and the combination of T36 and non-coating treatments took up the highest (15.15 days) and lowest (10.59 days) mean value of seedling emergence time, respectively (Figure 7).

#### Seedling root length

The variance analysis suggested that the inoculation treatments and coating significantly influenced the seedling root length of anise at different osmotic potential concentrations under drought stress. The comparison of mean values of the data revealed that an increase in drought stress would lead to a reduced root length of anise seedlings.

**FIGURE 8:**
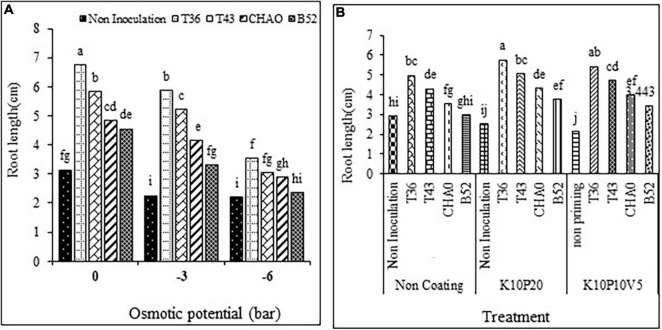
Mean comparison of the effect of seed inoculation on seedling root length (in cm) of anise at different levels of **(A)** drought stress and **(B)** coating. Means with the same letter are not significantly different according to the HSD test at *P* = 0.05. CHA0, B52 of *Pseudomonas fluorescens*, T36 and T43 of *Trichoderma harzianum* isolates. K, kaolin; V, vermiculite; P, perlite.

Among all the treatments, the highest root length was related to the T36 treatment, while the lowest root length was obtained with the non-inoculation treatment. Also, at each level of drought stress, the root length with the T36 treatment was longer than that with the other treatments (Figure 8A).

Comparing the interaction mean values of inoculation and the coating treatments showed that different inoculations with coating treatments significantly increased the seedling root length compared to the non-coating non-inoculation treatment. Among all the treatments, the highest value of root length (5.75 cm) was related to the T36 and K10P20 treatments (Figure 8B).

#### Seedling shoot length

**FIGURE 9:**
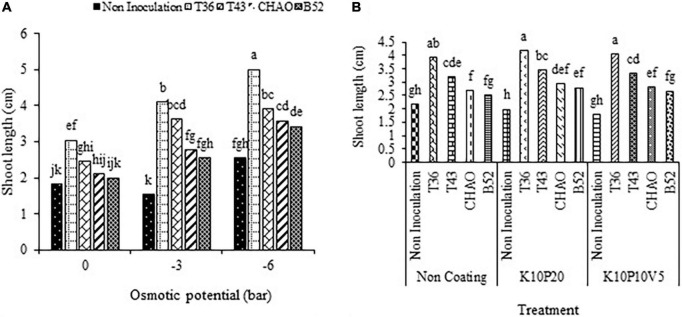
Mean comparison of the effect of seed inoculation on seedling shoot length (in cm) of anise at different levels of **(A)** drought stress and **(B)** coating treatments. Means with the same letter are not significantly different according to the HSD test at *P* = 0.05. CHA0, B52 of *Pseudomonas fluorescens*, T36 and T43 of *Trichoderma harzianum* isolates. K, kaolin; V, vermiculite; P, perlite.

Any increase in drought stress significantly increased the seedling shoot length but not the root length. The inoculation of seeds with bacterial and fungal treatments increased the shoot length of the seedlings at all potential osmotic levels compared to the non-inoculated seeds (Figure 9A). Comparing the mean values of different biological treatments at each level of drought stress showed that the lowest value belonged to the non-inoculation treatment. At the 0, –3, and –6 bars, the T36 treatment, increased the shoot length of the seedling by 67, 165, and 95.6%, respectively, compared to the non-inoculation treatment (Figure 9B). The results of the combination of biological and coating treatment revealed that the maximum shoot length (4.17 cm) was related to the T36 and K10P20 treatments, which lacked any significant difference with the combination of T36 and non-coating and the combination of the T36 and K10P10V5 treatments.

## Discussion

The results of the first experiment indicated that coating and biological treatments improved the physical properties, germination, and seedling indices of anise seeds in the laboratory. There was an increase in germination indices due to seed inoculation with fungal and bacterial biological treatments at all coating levels compared to the control seeds (non-inoculation). Among the superabsorbent coating treatments, the K10P20 treatment had higher germination percentage than the K10P10V5 treatment after the uncoated treatment. The increase in germination percentage of perlite coating treatments compared to kaolin and vermiculite can be attributed to the expansion ability and greater capacity of perlite to retain water around the seeds. Perlite can absorb and retain water three to seven times its weight, which facilitates hydration and gas exchange around seeds and improves germination (Howard, 2010).

Drought stress is a major problem in agricultural production. If it occurs in the germination stage, seedling growth will be reduced or even establishment will completely cease (Yan, 2015). In this experiment, the growth indices of the early seedlings’ of anise decreased with the increase in drought stress. The current results of this experiment are supported by the research findings that reported (Ahmed et al., 2014) drought stress reduces the growth parameters of sunflower seedlings. The reduction or lack of transfer of nutrients from cotyledons to seed embryos is one of the reasons for decreasing seedling length under drought stress. Besides, reduction in water uptake of seeds under stress reduces the activity of hormones and enzymes and, subsequently, disrupts the growth of seedlings (Voigt et al., 2009).

Seed coating can turn thin and fine seeds into larger spherical seeds and enhance the physical properties of seeds for proper and accurate planting. The present data in this study demonstrated that the addition of beneficial microorganisms, *Pseudomonas* bacteria and *Trichoderma* fungi, to seed coat increased the germination and seedling indices of anise in the laboratory and improved the initial vegetative growth of this plant under both optimal and drought stress and greenhouse conditions. The results of this study, indicated that seed coating with *Trichoderma* spp. and *Pseudomonas* spp. increased the germination percentage, germination rate, and seedling vigor indices under optimal and drought stress conditions (Moghaddam et al., 2018).

Research studies on *P. fluorescens*, *P. putsa*, and *Bacillus subtilis* found that these bacteria increased the germination indices of *Abieshickelii* and *Abiesreligiosa* seeds compared to untreated seeds. In the second experiment, the obtained findings are consistent with those of the study conducted on corn by Okoth et al. (2011). In similar research evaluating the effect of seed coating of sunflower with *P. putida* bacteria on sunflower indices under drought stress improved germination and seedling characteristics under drought stress were seen (Sandhya et al., 2009). In another related study, rice seeds coated with *P. fluorescence* SP700 s and kaolin-bearing bacteria had higher percentage and germination rate than control seeds. Indole-3-acetic acid-producing PGPR has improved cotton seeds’ germination and seedling length (Hafeez et al., 2004).

Bargamadi (2013) studied the influence of seed coating with vermiculite and *Trichoderma* on the growth and control of a disease of soybean, rhizoctonia. The coating of soybean seeds increased the percentage, germination rate, and seedling vigor of the seeds by combining biological treatments with *Trichoderma* fungi and vermiculite. Carriers such as vermiculite, perlite, talk, and kaolin used in seed coatings provide adequate moisture and porosity for the growth and viability of biological agents around seeds. Similarly, coating materials around seeds increase the vigor and viability of microbes by absorbing moisture, which protects the survival of microbes under environmental stresses like drought stress (Javed et al., 2022).

Inoculation of chickpea seeds with *T. viride* increased the roots and shoots of chickpeas. Likewise, the increase in roots and stem shoots by the bio-agent was due to the synthesis of the amino acid tryptophan, which is considered a precursor to auxin production (Kavusi et al., 2022). Different species of *Trichoderma* fungi increased the early growth and vegetation of seedlings under optimal and stress conditions with reduction of ethylene production arising from the reduced precursor of 1-aminocyclopropane 1-carboxylic acid (ACC) (Fazeli-Nasab and Sayyed, 2019; Fazeli-Nasab et al., 2022b) and auxin production (Contreras-Cornejo et al., 2009).

Bakhit and Moradi (2017) evaluated the impact of *Trichoderma* and *Pseudomonas* strains on flax seed germination and seedling indices. They found that the indices were increased significantly under storage conditions compared to control seeds. *Trichoderma* isolates increase root growth and plant development by producing growth-promoting hormones such as auxin, cytokine, and cytokine-like molecules like zeatin (Osiewacz, 2002). The present results demonstrate that the use of coating treatments without bioagents could only improve the physical quality of the seeds. However, the application of bioagents with superabsorbent treatments also enhanced the physiological parameters of anise seeds in the laboratory in addition to boosting their physical properties. Seed coating with a combination of a super-absorbent and a bioagent could partially mitigate the destructive effects of drought stress under greenhouse conditions.

## Conclusion

The findings of the experiments showed that low germination and poor establishment, especially in drought stress, are the main problems of anise seeds. The combination of biological treatments of *P. fluorescent* or *T. harzianum* and superabsorbent coating treatment could partly moderate the adverse effects of drought stress and improve the initial growth parameters of the seedlings. Overall, these data indicate that using the T36 + K10P20 treatment as the best treatment could enhance the initial vegetative growth of anise seedlings under optimal and drought stress conditions and improve the physical properties of anise seeds.

If fine seeds are small, inoculated, and coated, their physiological and physical properties will be improved for easy cultivation and proper establishment.

## Data availability statement

The original contributions presented in this study are included in the article/Supplementary material, further inquiries can be directed to the corresponding author/s.

## Author contributions

AS: conceptualization and supervision. AH: methodology and writing – original draft. RS, HB, AM, RP, BF-N, PP, MA, SO, and RD: writing – review and editing and formal analysis. PP and SO: funding acquisition. All authors contributed to the article and approved the submitted version.

## Conflict of interest

The authors declare that the research was conducted in the absence of any commercial or financial relationships that could be construed as a potential conflict of interest.

## Publisher’s note

All claims expressed in this article are solely those of the authors and do not necessarily represent those of their affiliated organizations, or those of the publisher, the editors and the reviewers. Any product that may be evaluated in this article, or claim that may be made by its manufacturer, is not guaranteed or endorsed by the publisher.
