# Supplementary figures and images for: Efficacy of biological agents and fillers seed coating in improving drought stress in anise
Source: Front Plant Sci. 2022 Jul 22;13:955512. doi: 10.3389/fpls.2022.955512 (PMC9355580; doi:10.3389/fpls.2022.955512)

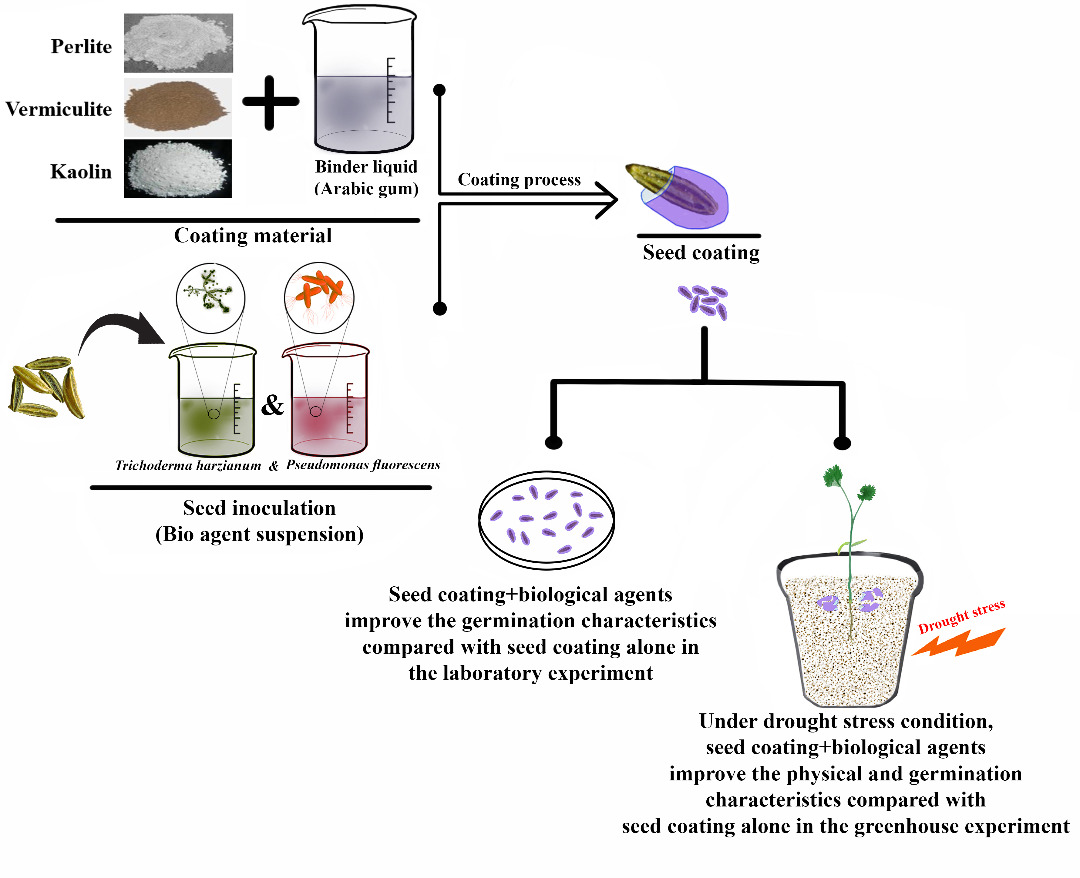

Supplement: Supplementary file 1 [file Image_1.JPEG]
